# Reduced bone formation and increased bone resorption drive bone loss in* Eimeria* infected broilers

**DOI:** 10.1038/s41598-023-27585-5

**Published:** 2023-01-12

**Authors:** Yuguo Hou Tompkins, Janghan Choi, Po-Yun Teng, Masayoshi Yamada, Toshie Sugiyama, Woo Kyun Kim

**Affiliations:** 1 Department of Poultry Science, University of Georgia, Athens, GA 30602 USA; 2 Graduate School of Science and Technology, Niigata University, 2-8050 Ikarashi, Nishi-ku, Niigata, 950-2181 Japan

**Keywords:** Animal biotechnology, Biotechnology

## Abstract

Coccidiosis is an economically significant disease in the global poultry industry, but little is known about the mechanisms of bone defects caused by coccidiosis; thus, the study focused on effects of coccidiosis on the bone homeostasis of young broiler chickens. A total of 480 male Cobb500 broilers were randomly allocated into four treatment groups, including an uninfected control consuming diet ad libitum, two infected groups were orally gavaged with two different concentrations of sporulated *Eimeria* oocysts, and an uninfected pair-fed group fed the same amount of feed as the high *Eimeria*-infected group consumed. Growth performance and feed intake were recorded, and samples were collected on 6 days post infection. Results indicated that coccidiosis increased systemic oxidative status and elevated immune response in bone marrow, suppressing bone growth rate (*P* < 0.05) and increasing bone resorption (*P* < 0.05) which led to lower bone mineral density (*P* < 0.05) and mineral content (*P* < 0.05) under *Eimeria* infection. With the same amount of feed intake, the uninfected pair-fed group showed a distinguished bone formation rate and bone resorption level compared with the *Eimeria* infected groups. In conclusion, inflammatory immune response and oxidative stress in broilers after *Eimeria* infection were closely associated with altered bone homeostasis, highlighting the role of inflammation and oxidative stress in broiler bone homeostasis during coccidiosis.

## Introduction

Coccidiosis induced by protozoan parasites, *Eimeria* spp., causes a sizeable economic impact in the poultry industry worldwide^1^. *Eimeria* spp. infect enterocytes and cause severe digestive tract damage, leading to inflammation and malabsorption of nutrients^2–4^. Coccidiosis affects chickens of all ages, although the negative impact of coccidiosis was more severe at younger ages owing to the immature immune system^5^. Furthermore, because the modern broiler chicken is characterized by relatively higher porosity and lower mineral content in long bones^6,7^, broiler chickens’ leg bones might be more frangible under the infection with *Eimeria* spp.^8–10^.

Coccidiosis significantly reduced tibia bone ash content and adversely affected femur breaking strength^11^. Sporozoites of some species, especially *E. acervulina* and *E. maxima*, also decreased bone mineral content (BMC) and showed lower bone mineral density (BMD) in infected broilers^12^. Bone mineral loss caused by *Eimeria* spp. in broiler chickens has been linked to nutrition imbalance and malabsorption with significantly reduced absorption of essential minerals and vitamins^13–17^. Other than the nutritional factors, emerging evidence suggests that bone homeostasis is mediated by oxidative stress and immune response under disease conditions^18,19^. It has been shown that oxidative stress is a negative impact factor of osteoblast activity in broilers during *Eimeria* infection, and there is a potential link between oxidative stress and lower bone quality^20^. Moreover, the activity of osteoclasts can be another factor to mediate skeletal homeostasis during pathogen infection^21,22^. Osteoclasts, unique multinuclear cells, originate from hematopoietic stem cells in bone marrow^23^ and are located on the surface of the bone^24^. Osteoclasts highly express tartrate-resistant acid phosphatase (TRAP, TRAPase)^25^. The expression and activity of the TRAP enzyme was not only considered as a histochemical biomarker of osteoclasts activity but also considered as an important messenger between skeleton homeostasis and the immune system^26^. Bone marrow also serves as the cradle of hematopoiesis and as a reservoir of growth factors and cytokines^27^. Many inflammatory cytokines trigger osteoclastic bone resorption by mediating osteoclast formation, cell activity, and lifespan, which ultimately leads to bone mineral loss^28–31^. The crosstalk between bone homeostasis and immunity was referred as osteoimmunology^32^. The receptor activator of nuclear factor kappa B (RANK) is essential in activating the nuclear factor kappa B (NFKB) pathway and the c-Fos (*FOS*, Fos proto-oncogene) pathway by mediating the expression of intracellular promoter nuclear factor of activated T cell (NFATC1) that stimulates the differentiation of osteoclasts^33–41^. NFKB ligand (RANKL) is a critical cytokine produced mainly in osteoblasts and regulates osteoclast formation^32,42^. The binding of RANKL to its receptor RANK on the surface of osteoclast progenitor cells triggers the differentiation of osteoclast precursors into osteoclasts, which increases the number of osteoclasts on their bone surfaces^43^. Besides, pro-osteoclastogenic cytokines produced by macrophages include tumor necrosis factor-α (TNF-α; tumor necrosis factor-α-like in chicken), interleukin-1β (IL1B), and interleukin-6 (IL6) are essential cytokines in mediating osteoclast activity^43–47^. Meanwhile, those cytokines are excessively released in response to *Eimeria* infection^48–50^. Moreover, several transcript factors have multiple roles in inflammatory response, osteogenesis, and osteoclastogenesis. For example, osteoprotegerin (OPG) has been shown to be an inhibitor of TNF-related apoptosis^51^. It is produced by osteoblast lineage cells and acts as a natural decoy receptor for RANKL, negatively regulating RANK-RANKL signaling and reducing bone resorption. The ratio of RANKL/OPG was used as a biomarker that indicates the occurrence of bone remodeling^52^. Modulating the expression of OPG/RANKL can affect the activity of osteoblasts, osteoclastogenesis, and osteoclast activity. SMAD1 is a key element that intermediates transforming growth factor-beta signaling and the bone morphogenetic protein (BMP) signaling pathways that are essential in osteoblast activity, bone mineralization, and osteoclast differentiation^53–55^.

In poultry studies, Kakhki et al.^56^ have reported *Eimeria* spp. had adverse effects on long bone homeostasis, which is attributed to bone remodeling status. However, the biomechanical properties of specific bone homeostasis and its precise etiology remain to be fully elucidated. Based on the aforementioned information, we hypothesized that broiler bone health during coccidiosis is not simply caused by nutritional deficiency but is also associated with inflammatory immune responses and oxidative stress in the broiler chicken. Thus, the study was conducted to better understand bone homeostasis in broiler chickens and the relationship between inflammation/oxidative stress and skeletal development during acute *Eimeria* infection.

## Results

### Performance variables and intestinal permeability

**Figure 1:**
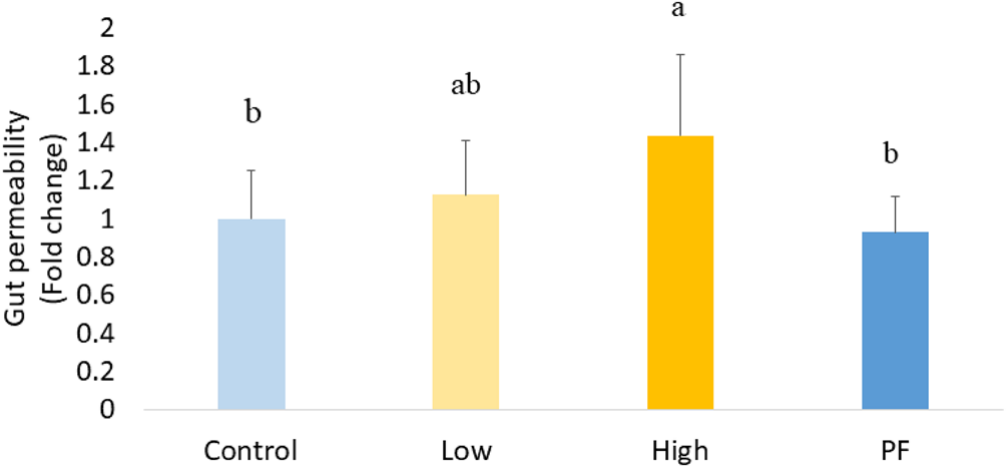
Gut permeability at 5 dpi. Higher concentration of FITC-d in the serum revealed more severe damage to the gut epithelium owing to the *Eimeria* challenge.^a,ab,b^ Treatments with different letters means a significantly difference between treatments by using Tukey’s HSD test, *P* < 0.05, N = 10. The relatively level of FITC-d in serum was presented as fold change.

On post-infection day 5 (5 dpi), the Control and the pair-fed (PF) groups showed no sign of intestinal damage, whereas a higher concentration of FITC-d in the serum was detected in the *Eimeria* challenged groups, which revealed damage to the gut epithelium owing to the *Eimeria* infection. Feed restriction in the PF group did not cause intestinal lesions. The high challenge group (High) had higher gastrointestinal permeability than the Control or PF (*P* < 0.05; Fig. 1). The average daily feed intake (ADFI) was recorded during the trial (Table 1). Because the same amount feed was provided to the PF to match with the High groups, there were no differences in ADFI between the PF and the High (*P* > 0.05). From 4 to 6 dpi, *Eimeria* infection significantly decreased ADFI in the Low (*P* < 0.05) and High (*P* < 0.05) groups when compared to the uninfected Control group. The result of the cumulative feed consumed (FI), body weight gain (BWG), and feed conversion ratio (FCR) clearly showed that *Eimeria* infection adversely affected all performance parameters (*P* < 0.05; Table 1). The low dose of *Eimeria* infection significantly reduced BWG and increased FCR (*P* < 0.05) in broilers compared with the Control during the whole acute challenge period (0–6 dpi); however, it did not significantly alter the FI during the infection. The high dose of *Eimeria* infection significantly reduced BWG (*P* < 0.05) and FI (*P* < 0.001), and increased FCR (*P* < 0.001) compared with the Control or Low group. Significantly lower BWG (*P* < 0.05), lower cumulative FI (*P* < 0.05), and higher FCR (*P* < 0.05) were observed in the PF group compared with the Control. Besides, the PF group showed a 16.21% higher BWG (*P* > 0.05) and significantly lower FCR (*P* < 0.05) when compared with the High.

**Table 1:** Average daily feed intake (ADFI), cumulative feed intake (FI), body weight gain (BWG, kg) and feed conversion ratio (FCR) from 0 to 6 dpi.

Treatments^1^	ADFI (g)					
	1 dpi	2 dpi	3 dpi	4 dpi	5 dpi	6 dpi
Control	126.01	95.42	104.23	108.19^a^	86.38^a^	96.68^a^
Low	134.75	103.97	107.01	86.89^b^	68.09^b^	64.51^b^
High	133.40	101.93	100.40	75.22^b^	42.18^c^	44.89^c^
PF	127.27	93.33	93.33	76.36^b^	42.42^c^	46.82^c^
SEM	1.66	2.32	2.16	2.92	3.56	3.86
*P*-value	0.158	0.316	0.128	< 0.001	< 0.001	< 0.001

Treatments	0–6 dpi					
	FI	BWG	FCR			
Control	617.75^a^	352.59^a^	1.77^c^			
Low	566.53^a^	250.43^b^	2.32^b^			
High	499.47^b^	178.06^c^	2.93^a^			
PF	480.38^b^	206.94^bc^	2.35^b^			
SEM	12.098	12.86	0.09			
*P*-value	< 0.001	< 0.001	< 0.001			

^1^Control: uninfected controls fed diet ad libitum and gavaged with water; Low: low *Eimeria-*infected group fed diet ad libitum and gavaged with 50,000 oocysts of *E. maxima*, 50,000 oocysts of *E. tenella*, and 250,000 oocysts of *E. acervulina*; High: severely *Eimeria*-infected group fed diet ad libitum diet and gavaged with 12,500 oocysts of *E. maxima*; 12,500 oocysts of *E. tenella*; 62,500 oocysts of *E. acervulina*; PF: an uninfected pair-fed group that fed the same amount of feed as the High group, gavaged with water.

^a,ab,b,c^Treatments with different letters means a significantly difference between treatments by using Tukey’s HSD test, *P* < 0.05, N = 10.

### Bone mineral density was reduced in the *Eimeria*-infected groups

Bone morphology parameters were analyzed in diaphysis and metaphysis sections using micro-computed tomography (micro-CT) (Table 2). Tibia diaphyseal BMC was significantly reduced in the High and PF groups (*P* < 0.05), and diaphyseal BMD was reduced in the Low (*P* < 0.05), High (*P* < 0.05) and PF groups (*P* < 0.05) compared with the Control. Tibia metaphyseal BMD (total) was significantly reduced in the Low (*P* < 0.05) and the High (*P* < 0.05), whereas there was no significant difference between the PF group (*P* > 0.05) and the Control. Trabecular BMD and the number of trabecular bone (Tb. N) at metaphyseal regions were significantly reduced in the Low, High, and PF groups (*P* < 0.05) compared to the Control. However, metaphyseal total BMC, metaphyseal trabecular BMC, metaphyseal cortical BMD, and metaphyseal cortical BMC were not affected by *Eimeria* infection or feed restriction.

**Table 2:** Femur bone metaphysis and diaphysis structure in broiler chickens at 6 dpi.

	Section		Unit	Control^1^	Low	High	PF	SEM	*P*-value
Metaphysis	Total	BMC^2^	g	149.403	137.218	117.639	132.312	6.984	0.469
		BMD	g/mm^2^	0.288^a^	0.233^b^	0.239^b^	0.243^ab^	0.009	0.017
		TV	mm^3^	579.742	606.012	534.340	559.222	22.068	0.704
		BV	mm^3^	315.092	359.677	301.793	335.881	20.723	0.783
	Cortical	BMD	g/mm^2^	0.556	0.511	0.522	0.492	0.0136	0.449
		BMC	g	84.366	97.495	86.034	95.740	4.9120	0.325
	Trabecular	BMD	g/mm^2^	0.519^a^	0.398^b^	0.392^b^	0.411^b^	0.0135	< 0.001
		BMC	g	4.339	4.286	3.304	5.483	0.4082	0.294
		BS	mm^2^	403.078	410.676	317.433	443.159	37.1790	0.660
		Tb.N	-	11.325^a^	9.1304^b^	9.236^b^	8.2574^b^	0.2855	0.004
Diaphysis	Total	BMC	g	136.838^a^	120.375^ab^	104.044^b^	96.318^b^	4.8538	0.013
		BMD	g/mm^2^	1.028^a^	0.902^b^	0.912^b^	0.898^b^	0.0130	< 0.001
		BV	Mm^3^	133.672	133.649	114.335	107.172	4.8897	0.126

^1^Control: uninfected controls fed diet ad libitum and gavaged with water; Low: low *Eimeria-*infected group fed diet ad libitum and gavaged with 50,000 oocysts of *E. maxima*, 50,000 oocysts of *E. tenella*, and 250,000 oocysts of *E. acervulina*; High: severely *Eimeria*-infected group fed diet ad libitum diet and gavaged with 12,500 oocysts of *E. maxima*; 12,500 oocysts of *E. tenella*; 62,500 oocysts of *E. acervulina*; PF: an uninfected pair-fed group that fed the same amount of feed as the High group, gavaged with water.

^2^BMC, bone mineral content; BMD, bone mineral density; TV, total bone volume; BV, bone volume (TV minus bone marrow volume); BS, bone surface area; Tb. N, trabecular number.

^a,ab,b^Treatments with different letters means a significantly difference between treatments by using Tukey’s HSD test, *P* < 0.05, N = 10.

### *Eimeria*-infected broilers exhibited suppression in bone formation

**Figure 2:**
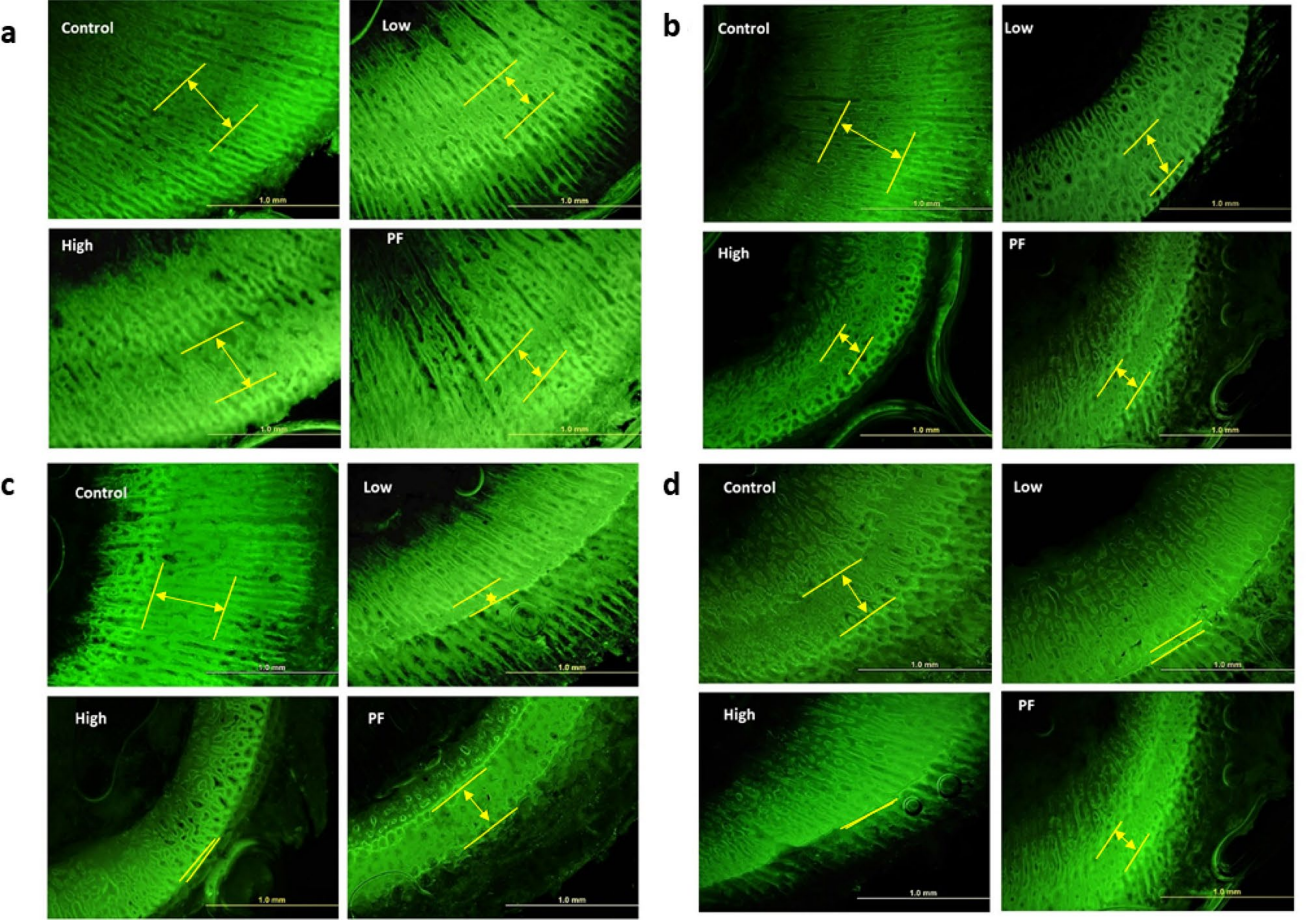
Fluorescence image of the femoral or tibial cross-section of broilers (measurement data shown as Table 3). Bone formation was visualized by double calcein labeling in the femoral bone and tibia bone, respectively. Representative picture (**a**): femoral bone at 0–4 dpi; (**b**): tibial bone at 0–4; (**c**): femoral bone at 4–8 dpi; (**d**): tibial bone at 4–8 dpi; Photos were taken at 4 × objective. The yellow scale bar is 1 mm in length. The yellow lines marked the distance between two calcein injections.

The distance between double layers of calcein bands was measured at the diaphysis of the tibia and femurs to evaluate bone growth rate (Fig. 2; Table 3). From 0 to 4 dpi (mild infection period), the femoral growth rate was significantly decreased in the High and PF groups compared to the Control, whereas the tibial growth rate was not significantly changed during 0–4 dpi. From 4 to 8 dpi (severe infection period), tibial and femoral growth was significantly reduced in the Low group (*P* < 0.05) and the High group (*P* < 0.05), however, it was not changed in the PF group (*P* > 0.05) when compared with the Control.

**Table 3:** Bone growth rate measured by calcein injection method.

Femur: unit (mm)			
Treatment^1^	0–4 dpi	4–8 dpi	0–8 dpi
Control	0.253^a^	0.212^a^	0.479^a^
Low	0.175^ab^	0.081^b^	0.219^bc^
High	0.157^b^	0.032^b^	0.190^c^
PF	0.145^b^	0.157^a^	0.300^b^
SEM	0.0132	0.0143	0.0241
P-value	0.014	< 0.001	< 0.001

Tibia: unit (mm)			
Treatment^1^	0–4 dpi	4–8 dpi	0–8 dpi
Control	0.208	0.186^a^	0.387^a^
Low	0.128	0.030^b^	0.147^b^
High	0.115	0.036^b^	0.152^b^
PF	0.125	0.140^a^	0.270^ab^
SEM	0.0177	0.0158	0.0285
P-value	0.197	< 0.001	0.002

^1^Control, control group; Low, the lower challenge dose; High, the high challenge dose; PF, pair-feeding group, paired with the High group.

^2^Unit: mm.

^a,ab,b,bc,c^Treatments with different letters means a significantly difference between treatments by using Tukey’s HSD test, *P* < 0.05, N = 10.

By adding data together from both injection stages, during 0–8 dpi, the Low, High, and PF treatment groups showed a significant decrease in bone formation (only femur), whereas the High group had the lowest bone growth rate (*P* < 0.05), followed by the Low (*P* < 0.05) and PF (*P* < 0.05) when compared with the Control. Compared with the Control, tibial growth in the Low and High groups was significantly reduced (*P* < 0.05) by the *Eimeria* challenge. However, the tibial growth of the PF group was not statistically different compared to the Control or infected groups (*P* > 0.05).

**Figure 3:**
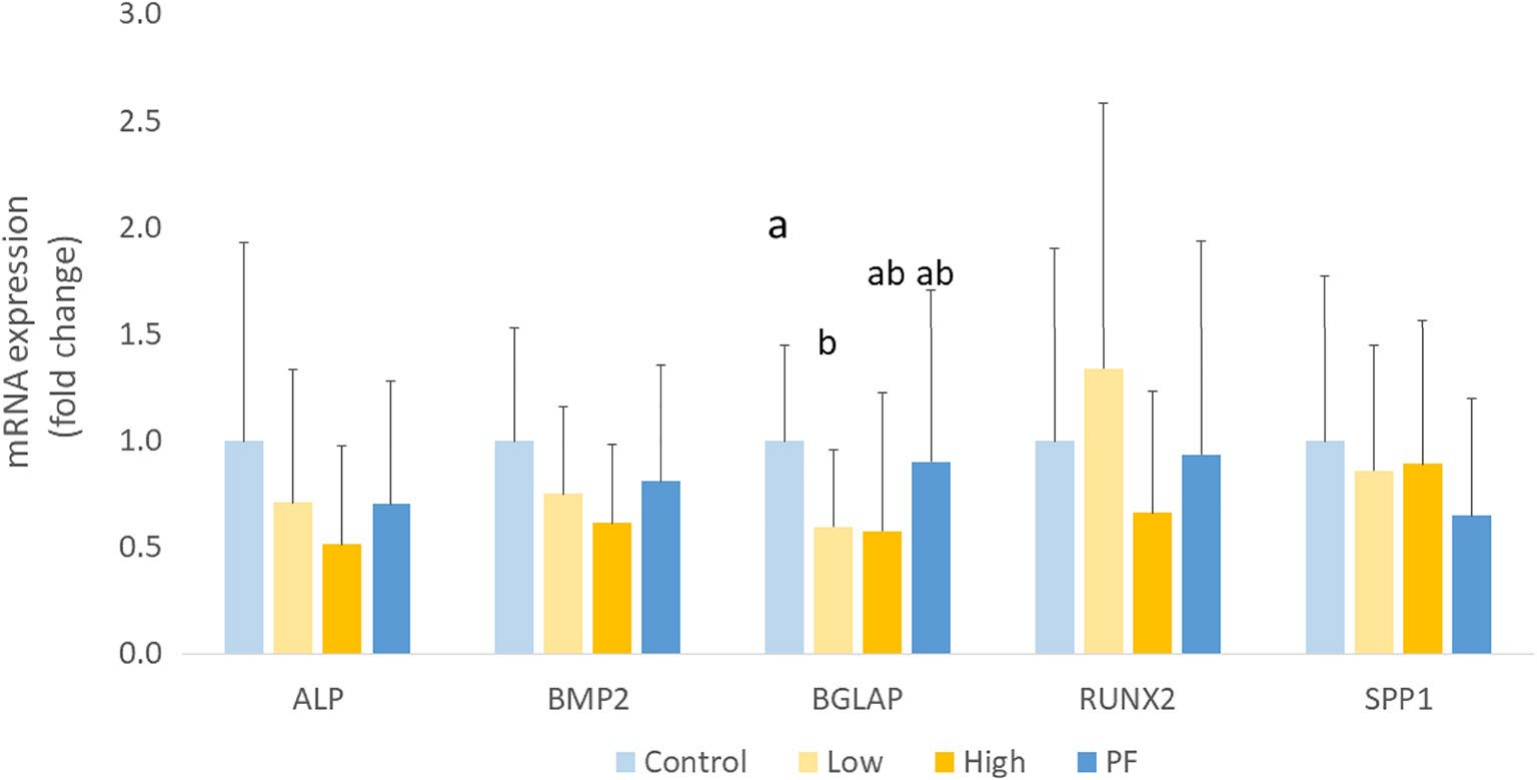
Osteogenesis-related gene expression in broilers bone marrow of different treatment groups. Control, non-challenge control group; Low, the low challenge dose of *Eimeria*; High, the high challenge dosage of *Eimeria*; PF, pair-feeding group that paired with High group.^a,ab,b^ Treatments with different letters means a significantly difference between treatments by using Tukey’s HSD test, *P* = 0.002, N = 10.

Relative mRNA expression of *BGLAP* was significantly reduced in the High group when compared with the Control (*P* < 0.05; Fig. 3), confirming that the severe infection of *Eimeria* adversely affected long bone growth. However, the expression of other bone formation markers was not significantly changed among the treatments. Moreover, higher cumulative FI was linearly correlated with higher femoral bone formation rate (R^2^ = 0.4655, *P* = 0.010), but not in tibia (*P* > 0.05). The tibial bone growth rate was highly correlated with metaphyseal trabecular BMD (R^2^ = 0.710, *P* < 0.001). Tibial bone growth rate (by calcein injection method) was positively correlated with tibial diaphysis BMD (R^2^ = 0.4308, *P* = 0.032).

### *Eimeria* infected broilers exhibited an increased bone resorption

**Figure 4:**
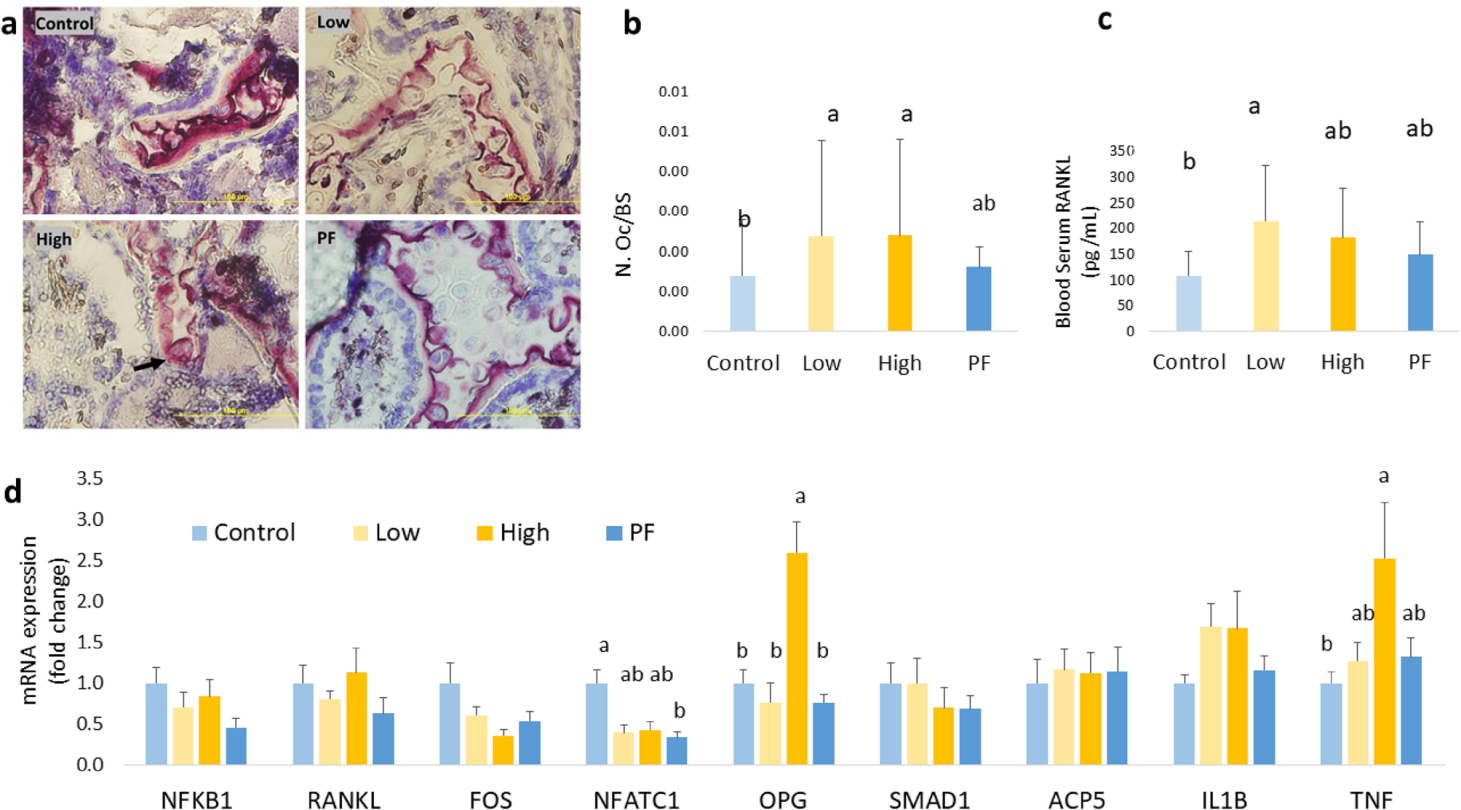
*Eimeria* infection increases osteoclast formation. (**a**): Illustration of TRAP staining at tibial metaphysis section. Osteoclasts were defined in this study as TRAP-positive cells with three or more nuclei. Osteoclast was indicated with arrow. Photos were taken at 10 × objective. The yellow scale bar is 100 µm in length. (**b**): Number of osteoclasts (N. Oc) were counted and bone circumference (BS) were measured. The increased ratio of N. Oc/BS indicated an increased formation of osteoclast activity at metaphysis section in *Eimeria* infected groups (*P* = 0.004). (**c**): Serum level of RANKL in broilers at 6 dpi. The low group has higher level of RANKL than the Control group (*P* = 0.047). (**d**): Osteoclastogenesis-related gene expression in broilers bone marrow of different treatment groups.^a,ab,b^ Treatments with different letters means a significantly difference between treatments by using Tukey’s HSD test, *P* < 0.050, N = 10. Control, non-challenge control group; Low, the low challenge dose of *Eimeria*; High, the high challenge dosage of *Eimeria*; PF, pair-feeding group that paired with High group.

**Figure 5:**
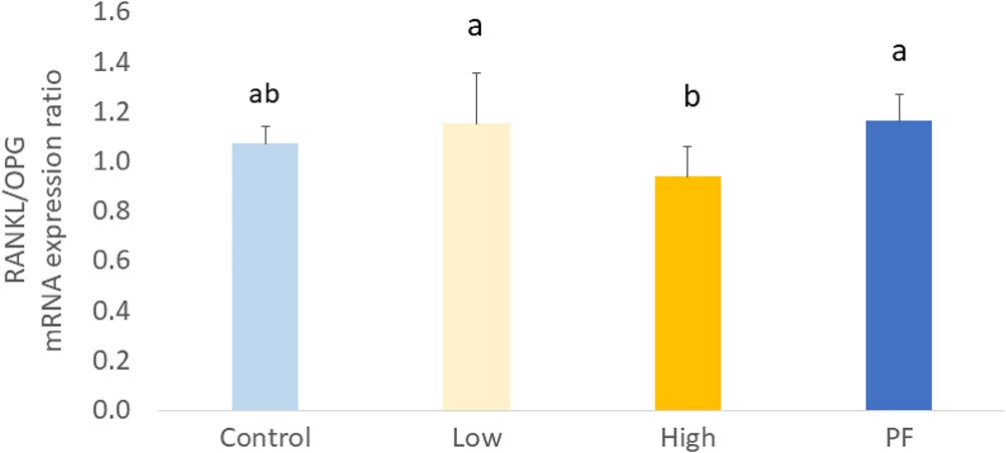
mRNA expression ratio of *RANKL*/*OPG* in bone marrow. Higher rate of *RANKL*/*OPG* indicated bone remodeling status.^a,ab,b^ Treatments with different letters means a significantly difference between treatments by using Tukey’s HSD test, *P* = 0.005, N = 10. Control, non-challenge control group; Low, the low challenge dose of *Eimeria*; High, the high challenge dosage of Eimeria; PF, pair-feeding group that paired with High group.

The number of osteoclasts per bone surface (N. Ocl/BS) in the Low and High groups was significantly higher than in the Control (*P* < 0.05; Fig. 4a and b). *Eimeria* challenge increased the formation of TRAP-positive cells and bone resorption activity on the surface of tibia metaphysis trabecular bone. Meanwhile, an increased serum level of RANKL was observed in the Low (*P* < 0.05; Fig. 4c), whereas the High and PF serum levels of RANKL had numeric increasing compared to the Control (*P* > 0.05). Moreover, the mRNA expression of *NFATC1* was significantly decreased in the PF (*P* < 0.05; Fig. 4d), and expression of *TNFRSF11B* (*OPG*) and *TNF* (tumor necrosis factor**-**like*)* was significantly increased in the High (*P* < 0.001) when compared with the Control. However, there were no significant changes in the expression of *NFKB1*, *RANKL*, *FOS*, *ACP5*(*TRAP*), *IL1B*, and *SMAD1* among the treatments. A higher ratio of *RANKL*/*OPG* was observed in the Low (*P* < 0.05) and the PF (*P* < 0.05) when compared with the High (Fig. 5). Together, results suggest that *Eimeria* infection resulted in bone remodeling along with higher osteoclast number and activity in broilers. There was a negative correlation between *TNF* and metaphyseal BMD (R^2^ = 0.419, *P* = 0.030).

### *Eimeria* infection increased lipid peroxidation and decreased antioxidant capacity, and the correlation between redox status and bone parameters

**Figure 6:**
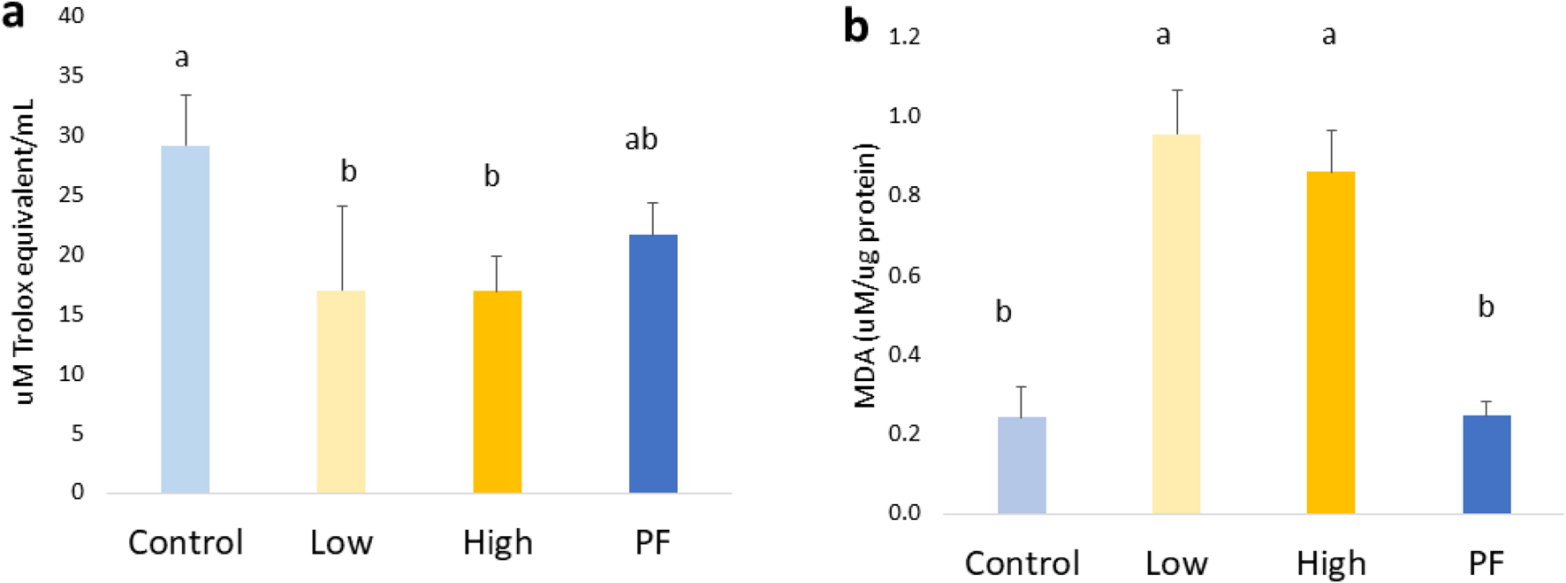
Oxidative status at 6 dpi. (**a**): serum total antioxidant capacity was measured by antioxidant assay kit, that coccidial infection significantly decreased the antioxidant capacity (*P* = 0.001). (**b**): liver lipid peroxidation was measured by TBAR assay kit, that coccidial infection significantly elevated lipid peroxidation in liver (*P* < 0.001). Control, non-challenge control group; Low, the low challenge dose of Eimeria; High, the high challenge dosage of Eimeria; PF, pair-feeding group that paired with High group.^a,ab,b^ Treatments with different letters means a significantly difference between treatments by using Tukey’s HSD test, *P* < 0.05, N = 10.

The total antioxidant capacity of serum in the *Eimeria spp*. infected broilers (the Low and High groups) decreased significantly at 6 dpi compared with the non-infected Control (*P* < 0.05; Fig. 6a). Meanwhile, the total antioxidant capacity of serum in the PF group showed a numeric decrease compared with the Control, but the change was not statistically significant. In contrast to the antioxidant parameters in serum, the level of MDA in the liver was significantly increased by *Eimeria* infection compared with the Control and PF groups (*P* < 0.01; Fig. 6b) at 6 dpi.

Pearson correlation analyses revealed a negative correlation of the liver TBAR level with femur growth rate (R^2^ = 0.249, *P* = 0.005) and between the liver TBAR level and tibia growth rate (R^2^ = 0.330, *P* = 0.001). There was a positive correlation between serum total antioxidant capacity and metaphysis bone mineral density (R^2^ = 0.219, *P* = 0.0182); and a negative correlation between the liver TBAR level and metaphysis bone mineral density (R^2^ = 0.391, *P* < 0.001). Meanwhile, bone marrow *BGLAP* mRNA level was negatively correlated with the liver TBAR level (R^2^ = 0.130, *P* = 0.031) and positively correlated with serum total antioxidant capacity (R^2^ = 0.227, *P* = 0.004).

## Discussion

With increasing concerns around farm animal welfare, poultry bone abnormalities have become one of the significant challenges for the poultry industry. Bone is an essential multifunctional organ that not only provides static functions such as structural support and internal organ protection but also acts as a dynamic endocrine organ that releases hormones for mineral homeostasis, acid–base balance, and reservoir of energy and minerals^57,58^. However, for a long time, bone health in broiler production yet gained enough attention until recently because more research suggests that current commercial broiler chicken breeds grow fast to heavyweight that predisposes the chickens to leg weakness and skeletal abnormalities^59,60^. With coccidiosis so widespread, the *Eimeria*-challenge model was chosen as a disease model to understand the possible link between intestinal parasite infection and bone health in the current study. With a mild infection, broilers was able to compensate for growth loss partially at later stage after recover from coccidiosis, even so, the growth potential remains severely compromised^48^. Uncoupling bone remodeling may not be apparent at early growth but may show up later in the market age, resulting in clinically leg bone abnormality, eventually decreasing the market value^61^. Thus, the early stage of bone health is vital in broiler growth performance and critical in product profitability.

The etiology of leg abnormalities under intestinal infection is generally complex. The factors that affect bone metabolism during infection include but are not limited to nutrition, immunity and physical stress. *Eimeria* infection could affect epithelium cells directly by mediating nutrition transporter activity or indirectly by causing apoptosis of cells and damaging the integrity of the intestine^3,62,63^. The damage to the digestive tract can cause malabsorption of nutrients, the deficiency of macronutrients including carbohydrates, crude protein, lipid, and minerals, and is associated with a bone remodeling imbalance that increases markers of bone resorption and decreases markers of bone formation^64–66^. Bone also serves as a mineral and energy reservoir, playing a role in maintaining glucose and phosphorus level within a narrow range in blood^67^. Long-term calcium deficiency is a potential risk factor for osteoporosis and bone fracture^68,69^. Depleting dietary calcium and phosphate could increase bone resorption and decrease bone mineral density^70,71^, but increased calcium intake alone is insufficient to compensate for the severe bone mineral loss under acute disease conditions^72^. Besides, protein deficiency could significantly decrease BMD and cancellous bone mass, reducing bone strength^13,14,73^. FI is positively correlated with the bone formation rate^74^. Lower energy, essential amino acid or mineral level can subsequently decrease osteoblastic activity suppressing bone formation. Moreover, nutrition deficiency could modify the plasma levels of certain essential hormones, such as growth hormone and insulin-like growth factor^75,76^, and elevated plasma stress hormones such as corticosterone^77–81^. Endocrine changes can directly mediate bone remodeling by regulating the activity of osteoblasts or osteoclasts, and those variables should also be considered in the experimental design. Monitoring the daily feed intake amount is very important for evaluating the growth performance especially with the impact of *Eimeria* infection on broiler chicken growth. In order to permit a clear interpretation of the results and limit the variables of nutrient and endocrine factors, the pair-feeding method was incorporated in this study. Pair-feeding is a technique to determine the effect of treatment on growth that is independent of nutritional factors. It has been widely used in the animal in vivo study model and was particularly well adapted to a study of energy, protein, and mineral intake deficiency^82,83^. In the current study, the amount of feed provided to the uninfected PF group was matched with the High group. However, the growth performance in the PF group was numerically higher than in the High group but not significant. The growth difference between the two groups reflected the metabolic cost of immune activation, oxidative stress, and other factors, rather than feed intake difference. Comparing the High group and the PF group provided more direct evidence to find the possible link between immune response/oxidative stress and bone metabolism.

Moreover, the micro-CT analyses revealed more specific details of bone structural changes. A significant drop in BMD was observed in bone metaphysis and diaphysis in both *Eimeria*-infected groups. Furthermore, the PF group and the infected Low and High groups showed different responses in the metaphysis and diaphysis sections. For example, the diaphysis is the mid-section (shaft) of a long bone and is the primary site of radial growth in young animals^84^. The diaphysis is made up of cortical bone, which has higher mineral content and density, and less water content when compared with trabecular bone, providing structural strength^85^. According to the current results, a significant decrease in BMD, BMC, and bone growth rate was observed in the diaphysis section. Both infected groups and the PF groups showed suppression of bone formation over this site. The PF group had the lowest diaphyseal BMC compared with the other groups. Lowest metaphyseal BMD was observed in the High group. It showed that bone metabolic activity appears site-specific during *Eimeria* infection, and bone mineral loss occurs distinctly in the proximal tibia metaphysis and diaphysis. Moreover, trabecular bones are metabolically more active than cortical bone, which not only contributes to the strength of the bone but also serves as a source of calcium for the body because trabecular bones are remodeled more rapidly during physiological processes^86–88^. Cortical bone loss occurs slower than trabecular loss due to the fact that less surface per unit of bone matrix volume is available for bone remodeling^89^. Nutrient deficiency, stress, and infectious skeletal disorders could cause bone mass loss and decrease bone quality by altering the trabecular bone microarchitecture^90,91^. In human and mouse research, the rate of bone turnover is more rapid in trabecular bone with a larger surface area than in cortical bone^92^. The current results showed that the bone mineral loss at the metaphysis section mainly occurred in the trabecular bone structure. Larger bone endo-surface could provide broader space for osteoblast or osteoclast attachment in trabecular bone. We hypothesized that bone resorption happens more frequently around the trabecular bone. Also, the tibial growth rate was highly correlated with tibial trabecular BMD instead of BMC, which indicates metaphysis trabecular bone BMD is more suited to evaluate early biochemical changes of bone during pathogen infection in broilers. Total mineral content (BMC) or bone ash weight may not accurately reflect the metabolic change of bone during infection.

Metabolic changes can be further assessed by different methods focusing on bone formation and resorption individually. Calcein labeling was used in the current study to visualize the newly formatted bone, and the RT-qPCR method was used to examine the bone formation-related marker genes in the bone marrow. The calcein labeling method is commonly used to assess bone growth, which directly visualizes bone growth in vivo. According to the results, by correlating FI with bone formation in each group, the Low group had significantly higher FI compared with the PF group; however, the bone formation rate was significantly lower than the PF (*P* < 0.05), which is in agreement with the findings of the microstructure analysis. With the same amount of FI, the PF group has a 4.71% higher BMD than the High group (*P* < 0.05). With a similar amount of FI (*P* > 0.05) between the Control and Low groups, diaphyseal BMD in the Low group was significantly lower than in the Control group. Based on the current results, even though provided with the same amount of feed, the *Eimeria*-challenged chickens had worse bone health status than the PF group, suggesting that apart from nutrition deficiency, other factors may be involved in bone homeostasis in both direct and indirect manners. In our previous studies, we have reported the changed redox status in broilers on 6 dpi after *Eimeria *spp. challenge^20^. Oxidative stress has been acknowledged as a major contributor to the immune response. The increased production of ROS is an inflammatory response that functions for the recruitment and activation of immune cells that lead to pathogen killing^93^. ROS production is involved in mineral homeostasis and contributes to bone remodeling by promoting bone resorption and suppressing bone formation. Human and mice studies have found a tight association between oxidative stress and pathogenesis of the bone disorder, that the redox state changes are related to the bone modeling and remodeling processes^94,95^. The redox state can directly impact osteoblast activity that regulates bone formation rate^96^. Oxidative stress suppressed the osteoblastic differentiation process of primary bone marrow stem cells^95^. In the current study, the oxidative stress (increased TBAR level and decreased total antioxidant capacity) was negatively correlated with bone growth rate and mRNA expression of *BGLAP*. This result was consistent with our previous finding^20^, that decreased bone quality was associated with systemic oxidative stress in broiler during *Eimeria* infection. Oxidative stress can be a co-factor involved in loss of osteoblastic activity that ultimately led to poor bone quality.

*Eimeria* infection can cause a complex host immune responses, encompassing both cellular and humoral mechanisms during infection^48^. Studies indicated that humoral immunity and antibodies produced by B cells were increased during severe *Eimeria* infection^97^. Different from other species, the bursa of Fabricius, a unique central immune organ of birds located near the cloaca, is the location of B lymphocyte differentiation and maturation instead of bone marrow^98^. Fully differentiated B lymphocytes migrate to peripheral lymphoid organs to participate in immune responses, such as producing antibodies and participating in humoral immunity^48,99^. B cell-produced proteins such as RANKL and OPG are critical for bone metabolism^100–104^. B lineage cells produce more than 60% of total OPG in bone marrow^102^. Mice that were injected with RANKL inhibitor resulted in a larger bone mass^105^. According to our study, an increased mRNA expression of *OPG* in bone marrow indicated that the system was actively producing more OPG, but the source of OPG remains unknown. The drastically higher expression of *OPG* in bone marrow may be related to a negative feedback loop, that increased *OPG* subsequently affects osteoclastogenesis^106^. We hypothesize that the bone marrow could actively reduce or inhibit highly-elevated osteoclast activity by increased expression of *OPG*, then preserving minerals for bone homeostasis during *Eimeria* infection. A similar pattern of expression was observed in gene expression of tight junction protein during acute *Eimeria* infection, that *Eimeria* infection damaged intestine integrity, but tight junction protein gene expression was significantly elevated to repair the damage^4^.

Bone marrow not only contains different cell types that perform bone formation and resorption but also serves as the cradle of hematopoiesis and a reservoir of growth factors and cytokines, providing an ideal environment for communicating between bone metabolism and the immune system. Essentially all the units that participate in cellular immunity can influence bone cells, particularly impacting the activity and formation of osteoclasts^2,97,107^. The cytokines IL-1β, IL-6, and TNF-α are known to increase bone resorption by stimulating both osteoclast activity and differentiation in mammals^45^. The number of osteoclast precursors increases under inflammatory conditions, characterized by high levels of the potent inflammatory cytokine TNF-α^108,109^. The current study found significantly higher mRNA expression of *TNF* (tumor necrosis factor***-***like) in the High group than in the other groups. Meanwhile, activation of osteoclast formation was detected in the High group over the metaphysis trabecular bone. It is important to mention that, with the same amount of FI, the expression of *TNF* was relatively low in the PF group compared with the High, as well as the number of osteoclast and enzyme activity of RANKL was relatively higher in the High group than the PF group. Based on the comparison between the High group and the PF group, we concluded that the increased osteoclastic bone resorption is associated with the activation of immune response in broiler chickens during *Eimeria* infection. Moreover, NFKB is involved in many signaling pathways and plays an important role in osteoclast formation and survival rate^35,110,111^. NFKB ligand (RANKL), one of the most critical molecules that regulate osteoclast formation, provides the crosstalk between bone and immune systems^32,42^. The binding of RANKL to its receptor RANK triggers osteoclast precursors to differentiate into osteoclasts, which increases the number of osteoclasts on their bone surfaces^43,112,113^. In the present study, the High group showed the lowest level of *RANKL*/*OPG*, indicating that the negative feedback loop was turned on because less osteoclastic activity was in need to preserve the minerals for bone structure and support^114^. The different expressions of the *RANKL*/*OPG* ratio between the Low and the High indicated the bone homeostasis is infection-dose-dependent during coccidiosis. However, with the difference in B-cell development in avian species, immunity, particularly humoral immunity, might interact with bone metabolism differently from mammals. How osteoimmunology plays a role in avian bone homeostasis needs more profound studies. Taken together, delayed bone development in the parasite-challenged groups was attributable to an uncoupling of osteoblast and osteoclast activity, whereby increased bone resorption and decreased bone formation were closely associated with immune response/oxidative stress during *Eimeria* infection. With the long-held notion that the central pathophysiology of bone disorder was nutrition deficiency and physical stress during *Eimeria* infection, we demonstrated that bone disorder is also closely connected with bone modeling and remodeling which are associated with immune response/oxidative stress. Both nutrition and concurrent diseases will influence the occurrence of leg disorders. Further study on osteoimmunology needs to address bone disorder issues and will further lead to a more precise understanding of the mechanism underlying the pathogenesis of bone mineral loss and bone disease in broilers, eventually improving animal production and welfare in the future.

## Materials and methods

### Ethics statement

The experiment followed the guideline of the Institutional Animal Care and Use Committee and was conducted at the Poultry Research Farm, University of Georgia, Athens, GA. The protocol was approved by the Institutional Animal Care and Use Committee at the University of Georgia (ethical approval code: A2021 12-012).

### Experimental design

The study was carried out in compliance with the ARRIVE guidelines. A total of 480 one-day-old male broiler chickens (Cobb 500) were randomly distributed into four treatment groups with ten replicates and twelve birds per cage. All broiler chicks were fed the same starter basal diet during day 1 to 14, and the starter diet were formulated following Cobb500 broiler management guide^115^. On day 14, all experimental groups received either water or *Eimeria* spp. challenge. Experimental groups included uninfected controls (Control) fed diet ad libitum (gavaged with water), a low *Eimeria-*infected group (Low) fed diet ad libitum (gavaged with 50,000 oocysts of *E. maxima*, 50,000 oocysts of *E. tenella*, and 250,000 oocysts of *E. acervulina*), a severely *Eimeria*-infected group (High) fed diet ad libitum diet (gavaged with 12,500 oocysts of *E. maxima*; 12,500 oocysts of *E. tenella*; 62,500 oocysts of *E. acervulina*), and an uninfected pair-fed group (PF; gavaged with water) that fed the same amount of feed as the High group consumed. To ensure that the pair-fed group (PF) had the same intake as the high challenge group, the amount of feed provided to each group was carefully monitored and matched. Preliminary data was used to estimate the daily feed intake of each group, and the average feed intake of the high challenge group was calculated. The same amount of feed was then provided to the pair-fed group. The feed was weighed and distributed evenly to the pair-fed group broilers at three or four intervals throughout the day (7:30 am, 3:30 pm, and 9:30 pm), with the 9:30 pm feeding time serving as an opportunity to adjust the intake of the pair-fed group to match that of the high challenge group. The grower (15–20 days of age) basal diets were formulated following Cobb500 broiler management guide^115^. Diet information is shown in Supplementary Table S1 online. All chicks were raised under the same house, feeding, and environmental management conditions based on the broiler management guide^115^. Chicks were allowed to consume water on an ad libitum basis, and daily feed intake was measured during the study. On 6 days post infection (dpi), one bird per replicate was randomly selected to collect tissue samples. The experimental design flow chart shown in Supplementary Fig. S1 online. The tissue samples were snap-frozen in liquid nitrogen and kept in − 80 °C until future processing.

### Gut permeability

The gut permeability was measured on 5 dpi by the method used in our previous study^3,116^. Briefly, fluorescein isothiocyanate dextran (FITC-d; MW 4000; Sigma-Aldrich, Ontario, Canada) was dissolved in distilled water and made into 2.2 mg/mL solution. One bird per cage was randomly selected and gavaged with 1 mL of FITC-d solution. Two hours after inoculation, the blood was collected from birds and kept in the dark at room temperature for clotting. The clotted blood was centrifuged at 1500 g for 15 min to serum collection. The standard curve solution was made from a serial dilution of FITC-d stock (2.2 mg/mL). Dilution buffer was made from the pooled serum of non-infection birds with the basal diet. Sample and standard solutions were loaded into black 96-well plates, and FITC-d concentrations were measured by a spectrophotometer (SpectraMax M5; Molecular Devices, San Jose, CA). The excitation wavelength was set at 485 nm, and the emission wavelength was set at 528 nm.

### Micro-computed tomography (micro-CT)

A total of 40 samples (one bird per cage) were randomly collected to evaluate 3-D bone morphologic changes in the broiler. The proximal and shaft of the tibia were assessed by micro-computed tomography (micro-CT). The scanning process was performed according to our previous publications^20,117^, with setting as 83 kV, 121 µA, and a 0.5 mm aluminum filter, the pixel size as 26 µm with 360° complete rotation, and 42 min of acquisition time. Scanning was performed with SkyScan 1172 (SkyScan, Kontich, Belgium). 2-D images were transferred to CTAn software (CTAn, SkyScan) for structure construction and quantification. The metaphyseal region of interest (ROI) was post-operated to automatically separate trabecular bone from cortical bone and preserve its morphology using a threshold of 800. Average bone mineral density (BMD), bone mineral content (BMC), and bone micro-architectural parameters of each group were taken from the same ROI. Cortical and trabecular bone parameters were quantified and analyzed separately. The following parameters were quantified: total volume (TV), bone volume (BV), bone surface (BS), bone volume per tissue volume (BV/ TV), and trabecular number (Tb. N)^20^.

### Calcein labeling

For dynamic histomorphometry measures of bone formation, calcein (Cat no. C0875, Sigma Aldrich, St. Louis, MO) was dissolved in a 1 M sodium hydroxide solution and then mixed with sterilized distilled water to make the 2.0% working solution. The birds were injected with the calcein solution intraperitoneally at 20 mg/kg of body weight. On day 4 after the first injection of calcein, the birds were injected again as previously described. Bone samples were collected on 4 days after the second injection. The muscle was removed immediately, and bones were preserved in 70% ethanol. Upon analysis, a thin slice of bone was taken from mid-diaphysis by a circular saw (Ryobi, Anderson, SC, USA), sanded each bone slice down by using sandpaper then mounted on a glass slide. Calcein has a high calcium affinity and translates into a relatively broad fluorescent band*.* A fluorescence microscope (Leica DC500 camera, Leica Microsystems Inc., Buffalo Groove, IL) was used to visualize new bone formation and determine the distance between the two calcein labels on the bones. Eight measurements at different angles were performed using ImageJ software (National Institutes of Health, Bethesda, MD, USA). The average values were calculated for data analysis.

### Serum receptor activator of nuclear factor kappa B ligand enzyme-linked immunosorbent assay (RANKL ELISA)

Chicken RANKL concentrations were measured by commercially available kits (MyBioSource, San Diego, CA, USA). All procedures were performed according to the manufacturer’s protocol. The method was two-site sandwich ELISA, the pre-coated antibody was Chicken PRM1 monoclonal antibody and the detecting antibody was polyclonal antibody with biotin labeled. A standard curve was created, and the RANKL concentration of the examined samples was calculated and expressed in pg/ml. Background OD values were subtracted from the calculation, and the color depth was directly proportional to the amount of RANKL in the sample.

### Tartrate-resistant acid phosphatase staining (TRAP staining)

All tibia bones were collected at 6 dpi. After removing the muscle tissue, tibias were fixed in 4% PBS-buffered formaldehyde at 4 °C for three days and then moved into 70% ethanol for preservation. Tibial tuberosity was used as a landmark to cut the bone slides by the circular saw. The bone slides were demineralized with 10% ethylenediaminetetraacetic acid (EDTA) at 4 °C for 13 days. Each bone slide was equally cut into four pieces perpendicularly. The samples were then embedded in paraffin and cut into 4 µm sections using Leitz 1512 rotary microtome (Leica Microsystems, Wetzlar, Germany). The paraffin sections were stained with tartrate-resistant acid phosphatase (TRAP) solution prepared by mixing acetate buffer (pH 5.0), naphthol AS-MX phosphate (Sigma Chemical, St. Louis, MO, USA), Fast Red Violet LB Salt (Sigma), and 50 mM sodium tartrate (Sigma). The sections were counterstained with hematoxylin (Sigma). Osteoclasts were defined in this study as TRAP-positive cells with three or more nuclei. Bone circumference was measured using ImageJ software (National Institutes of Health, Bethesda, MD, USA).

### Lipid peroxidation and antioxidant status assay

Chicken total antioxidant capacity in serum was analyzed using a QuantiChrom antioxidant assay kit (BioAssay Systems, Hayward, CA, USA), and the level of liver lipid peroxidation was determinated by using QuantiChro TBARS Assay Kit (BioAssay Systems, Hayward, CA, USA). Serum was collected, centrifuged, and then kept at − 80 °C. A liver sample from one bird per cage was collected, snap-frozen in liquid nitrogen, and then kept at − 80 °C. Liver samples were homogenized and centrifuged in the assay buffer, and all assay procedures were performed according to the manufacturer’s protocols. The protein concentration was measured by protein quantification assay (Pierce™ BCA Protein Assay Kit, Thermo Scientific, Rockford, IL, United States) following the procedure indicated in our previous publication^20^.

### Real-time quantitative PCR analysis of gene expression in bone marrow

Bone marrow from femur bones was extracted, snap-frozen in liquid nitrogen, and stored immediately at -80 °C until RNA isolation (N = 10). Bone marrow total RNA was extracted using Qiazol reagent (Qiagen, USA) according to the manufacturer’s instruction. A Nano-Drop 1000 Spectrophotometer (ThermoFisher Scientific, Pittsburgh, PA) was used to determine the quantity of RNA. The cDNA was synthesized from total RNA (2000 ng) using high-capacity cDNA reverse transcription kits (Thermo Fisher Scientific, Waltham, MA).

Real-time reverse transcription polymerase chain reaction (RT-qPCR) was performed to measure mRNA expression. Primers were designed using the Primer-BLAST program (https://www.ncbi.nlm.nih.gov/tools/primer-blast/). The specificity of primers was validated by melting curve analysis and gel electrophoresis. RT-qPCR was performed on an Applied Biosystems StepOnePlus™ (Thermo Fisher Scientific, Waltham, MA) with iTaq™ Universal SYBR Green Supermix (BioRad, Hercules, CA) using the following conditions for all genes: 95 °C for 10 min followed by 40 cycles at 95 °C for 15 s, annealing temperature for 20 s, and extending at 72 °C for 1 min.

The geometric mean of *18S*, *HBMS* and *GAPDH* were used for normalization^118^. The stability of housekeeping genes were confirmed by their consistent Ct values among the treatments (*P* > 0.1)^119^. *BGLAP*, *RUNX2*, *SPP1*, *BMP2*, and *ALP* were used as genetic markers of bone formation in the bone marrow^120^. *NFKB, RANKL, FOS, ACP5, NFATC1, IL1B, TNF, SMAD1*, and *TNFRSF11B* (*OPG*)^121^ were used as the genetic markers for osteoclastic activity in the bone marrow. Details of primer sequences used for the experiment are presented in Table 4. Moreover, the ratio of *OPG*/*RANKL* in bone marrow was calculated. Samples were run in triplicate, and relative gene expression data were analyzed using the 2^−ΔΔCt^. The mean ΔCt of each marker gene from the control group was used to calculate the ΔΔCt value, and 2^−ΔΔCt^ expression levels were normalized to 1 for the control group. Expression levels of the treatment groups were presented as fold change.

**Table 4:** Nucleotide sequences of the primers used for real-time RT-PCR.

	Primer sequence (5′–3′)	Product length (bp)	Annealing temperature (°C)	Accession #
GAPDH	F-GCTAAGGCTGTGGGGAAAGT R-TCAGCAGCAGCCTTCACTAC	161	55	NM_204305.1
RNA18S1	F-AGCCTGCGGCTTAATTTGAC R-CAACTAAGAACGGCCATGCA	121	56.5	AF_173612.1
HMBS	F- GGCTGGGAGAATCGCATAGG R- TCCTGCAGGGCAGATACCAT	131	59	XM_004947916.3
SPP1	F-GCCCAACATCAGAGCGTAGA R-ACGGGTGACCTCGTTGTTTT	204	57	NM_204535.4
BMP2	F-TCAGCTCAGGCCGTTGTTAG R-GTCATTCCACCCCACGTCAT	163	57	XM_025148488.1
RUNX2	F-ACTTTGACAATAACTGTCCT R-GACCCCTACTCTCATACTGG	192	60	XM_015285081.2
ALP	F-CGACCACTCACACGTCTTCA R-CGATCTTATAGCCAGGGCCG	140	58	NM_205360.1
BGLAP	F-GGATGCTCGCAGTGCTAAAG R-CTCACACACCTCTCGTTGGG	142	57	NM_205387.3
NFKB1	F-GAAGGAATCGTACCGGGAACA R-CTCAGAGGGCCTTGTGACAGTAA	131	59	XM_015285418.2
FOS	F-CTTCGACGAGCTGCTTTTCT R-TGGAGGTGTAGGTGCTAGGG	191	60	NM_205508.1
TNFRSF11B	F-ACGCTTGTGCTCTTGGACAT R-CAGCGTAGTACTGGTCTGGG	193	60	NM_001033641.1
TNFSF11	F-ACACGCCCTTTGAAAATCAG R-GCAAAAGGTTGCTTCTCTGG	196	60	XM_015275777.2
ACP5	F-GCTTCCAGGAGACCTTCGAG R-CAGGCGGAGGCTGTAGTAGT	170	61	XM_040693093.1
NFATC1	F-CAGTCCTGCAGTCCAACTCA R-TCCTCAGGTTCTCGCTTGAT	173	60	XM_040663226.1
SMAD1	F-GTTTTGTAAAGGGTTGGGGAGC R-AATGCAGGAGCTTGGGACCTTA	174	61	XM_040698719.1
IL1B	F-AGATGAAGCGGGTCAGCTC R-GCATCAAGGGCTACAAGCTC	120	59	XM_015297469.2
TNF	F-CGTGGTTCGAGTCGCTGTAT R-CCGTGCAGGTCGAGGTAC	100	60	XM_040694846.2

^1^GAPDH: glyceraldehyde-3-phosphate dehydrogenase; HMBS: hydroxymethylbilane synthase; RNA18S1: RNA, 18S ribosomal 1; ACP5: TRAP, acid phosphatase 5, tartrate resistant; TNFSF11: RANKL,TNF superfamily member 11; TNF: tumor necrosis factor-like; NFATC1:nuclear factor of activated T cells 1; TNFRSF11B: OPG, TNF receptor superfamily member 11b; IL1B: interleukin 1 beta; BGLAP: bone gamma-carboxyglutamate protein; RUNX2: runt-related transcription factor 2; ALP: alkaline phosphatase; SPP1: secreted phosphoprotein 1; BMP2: bone morphogenetic protein 2; FOS: Fos proto-oncogene, AP-1 transcription factor subunit; NFKB1: nuclear factor kappa B subunit 1; SMAD1: SMAD family member 1.

### Statistical analysis

All experimental data were expressed as mean with standard error of the means (SEM). Data were tested for homogeneity of variances and normality of studentized residuals. The differences among the treatment groups were analyzed by one-way ANOVA, whereas the means were analyzed statistically by Tukey’s test using JMP Pro14 (SAS Institute, Inc., Cary, NC). Statistical significance was set at *P* < 0.05, and 0.05 ≤ *P* ≤ 0.1 were also presented to show the trending toward statistical significance^122^. Pair wise correlations (JMP Pro14) were evaluated for all bone and growth variables.

## Supplementary Information

Supplementary Information.

## Data Availability

The datasets generated during the current study are available from the corresponding author on reasonable request.
