# Supplementary material for: Reduced bone formation and increased bone resorption drive bone loss in Eimeria infected broilers
Source: Sci Rep. 2023 Jan 12;13:616. doi: 10.1038/s41598-023-27585-5 (PMC9837181; doi:10.1038/s41598-023-27585-5)
Supplement: Supplementary file 1 — Supplementary Information. [file 41598_2023_27585_MOESM1_ESM.pdf]

Supplementary information

# Reduced bone formation and increased bone resorption drive bone loss in *Eimeria* infected broilers

Yuguo Hou Tompkins, Janghan Choi, Po-Yun Teng, Masayoshi Yamada, Toshie Sugiyama, Woo Kyun Kim*

Supplementary Table S1: Composition and calculated nutrient content of basal diets.

| Item | 0-14 d | 15-21 d |
|---|---|---|
| Ingredients, % | | |
| Corn, Grain | 65.20 | 70.08 |
| Soybean meal -48% | 29.49 | 24.17 |
| Dical. Phos. | 1.58 | 1.40 |
| Soybean oil | 0.79 | 1.85 |
| Limestone | 0.72 | 0.61 |
| Common salt | 0.15 | 0.18 |
| DL-methionine | 0.32 | 0.29 |
| Vitamin Premix[1] | 0.25 | 0.25 |
| L-lysine-HCL | 0.30 | 0.28 |
| Threonine | 0.12 | 0.08 |
| Mineral premix[2] | 0.08 | 0.08 |
| Sand | 1.00 | 1.00 |
| | | |
| Calculated energy and nutrient composition | | |
| ME, kcal/kg | 3008 | 3086 |
| Crude protein % | 21.00 | 19.00 |
| Dig-Lysine % | 1.18 | 1.05 |
| Dig-Methionine % | 0.59 | 0.53 |
| Dig-TSAA % | 0.88 | 0.80 |
| Dig-Threonine % | 0.77 | 0.69 |
| Ca % | 0.90 | 0.84 |
| avP % | 0.45 | 0.42 |

[1]Vitamin premix include Vit. A 2,204,586 IU, Vit. D3 200,000 ICU, Vit. E 2,000 IU, Vit. B12 2 mg, Biotin 20 mg, Menadione 200 mg, Thiamine 400 mg, Riboflavin 800 mg, d-Pantothenic Acid 2,000 mg, Vit. B6 400 mg, Niacin 8,000 mg, Folic Acid 100 mg, Choline 34,720 mg.

[2]Mineral premix include Ca 0.72 g, Mn 3.04 g, Zn 2.43 g, Mg 0.61 g, Fe 0.59 g, Cu 22.68 g, I 22.68 g, Se 9.07 g.

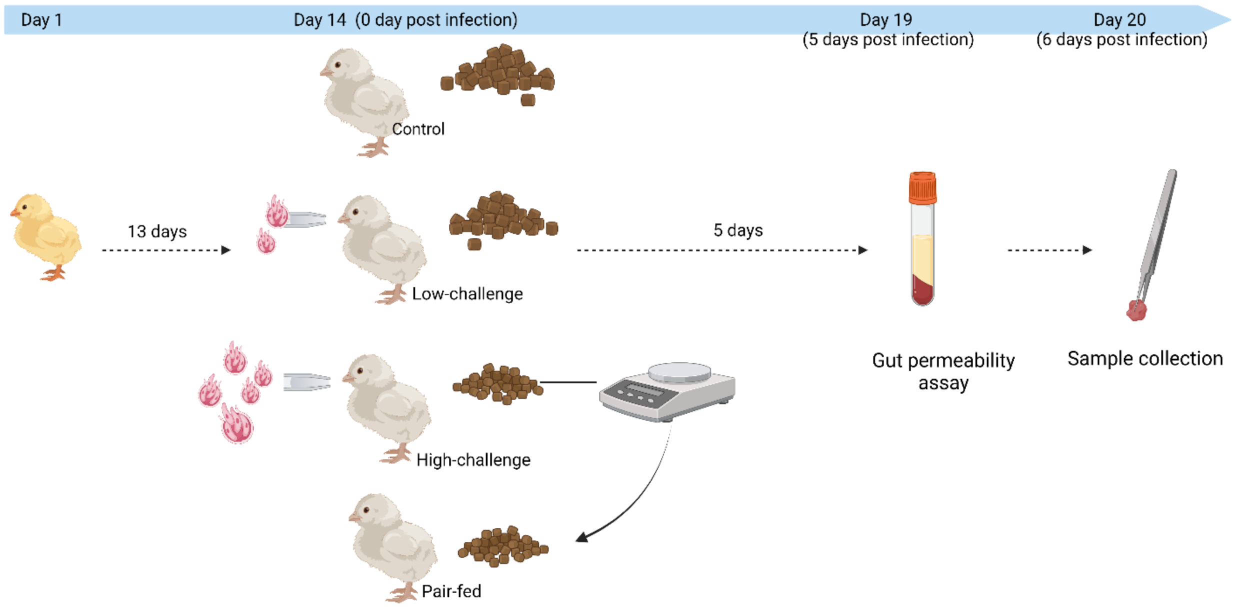


Supplementary Figure S1: The overview of the experiment design workflow. Created with BioRender.com.

Broiler chicks were raised at the same environment until day 14. All broiler chicks were fed with the same starter basal diet during day 1 to day 14. On day 14, all experimental groups received either water or *Eimeria spp.* challenge. Experimental groups included uninfected controls (Control) fed diet *ad libitum* (gavaged with water), a low *Eimeria*-infected group (Low) fed diet *ad libitum* (gavaged with 50,000 oocysts of *E. maxima*, 50,000 oocysts of *E. tenella*, and 250,000 oocysts of *E. acervulina*), a severely *Eimeria*-infected group (High) fed diet *ad libitum* diet (gavaged with 12,500 oocysts of *E. maxima*; 12,500 oocysts of *E. tenella*; 62,500 oocysts of *E. acervulina*), and an uninfected pair-fed group (PF; gavaged with water) that fed the same amount of feed as the High group consumed. The High challenge group feed intake amount were measured three times per day, and the same amount of feed were evenly distributed to PF group throughout the day to match with the High group. On 6 days post infection (dpi), one bird per replicate was randomly selected to collect tissue samples.
